# Effect of low-load resistance training with different degrees of blood flow restriction in patients with knee osteoarthritis: study protocol for a randomized trial

**DOI:** 10.1186/s13063-021-05946-7

**Published:** 2022-01-03

**Authors:** Hao-Nan Wang, Yan Chen, Lin Cheng, Shen-Tao Wang, De-Xin Hu, Li-Na Wang, Guo-Xin Ni

**Affiliations:** School of Sport Medicine and Rehabilitation, Beijing Sport University, No. 48 Xinxi Road, Haidian District, Beijing, 100084 China

**Keywords:** Knee osteoarthritis, Blood flow restriction, Vascular occlusion, Resistance training, Rehabilitation

## Abstract

**Background:**

Knee osteoarthritis (KOA) is a common degenerative disease that causes pain, functional impairment, and reduced quality of life. Resistance training is considered as an effective approach to reduce the risk of muscle weakness in patients with KOA. Blood flow restriction (BFR) with low-load resistance training has better clinical outcomes than low-load resistance training alone. However, the degree of BFR which works more effectively with low-load resistance training has not been determined. The purpose of this study is to evaluate the effectiveness of different degrees of BFR with low-load resistance training in patients with KOA on pain, self-reported function, physical function performance, muscle strength, muscle thickness, and quality of life.

**Methods:**

This is a study protocol for a randomized, controlled trial with blinded participants. One hundred individuals will be indiscriminately assigned into the following groups: two training groups with a BFR at 40% and 80% limb occlusion pressure (LOP), a training group without BFR, and a health education group. The three intervention groups will perform strength training for the quadriceps muscles twice a week for 12 weeks, while the health education group will attend sessions once a week for 12 weeks. The primary outcome is pain. The secondary outcomes include self-reported function, physical function performance, muscle strength of the knee extensors, muscle mass of the quadriceps, quality of life, and adverse events. Intention-to-treat analysis will be conducted for individuals who withdraw during the trial.

**Discussion:**

Previous studies have shown that BFR with low-load resistance training is more effective than low-load resistance training alone; however, a high degree of BFR may cause discomfort during training. If a 40% LOP for BFR could produce similar clinical outcomes as an 80% LOP for BFR, resistance training with a low degree of BFR can be chosen for patients with KOA who are unbearable for a high degree of BFR.

**Trial registration:**

Chinese Clinical Trial Registry ChiCTR2000037859 (http://www.chictr.org.cn/edit.aspx?pid=59956&htm=4). Registered on 2 September 2020

## Background

Knee osteoarthritis (KOA) is one of the most common chronic musculoskeletal diseases. It can develop the deterioration in the subchondral bone, cartilage, synovium, and menisci [1], resulting in pain, stiffness, impaired functioning in daily activities, and a decline in the quality of life (QoL). According to an epidemiological survey, it is estimated that the prevalence of symptomatic radiographic KOA among older people is 12.1% in the USA [2] and 8.1% in China [3], and these figures are manifesting an upward trajectory. Due to its high prevalence, KOA may cause tremendous economic burdens on health services, such as conventional interventions and joint replacements [4]. Therefore, primary and secondary prevention programs are necessary to minimize social and personal costs.

Multiple risk factors may contribute to the development of KOA, such as age, sex, obesity, and joint factors; unfortunately, the link between these factors and KOA remains unclear [5–7]. Nevertheless, muscle weakness, especially in the quadriceps, is considered both a vital and a modifiable risk factor for KOA [8]. Previous studies have shown that muscle weakness is strongly associated with the incidence [8] and progression [9] of KOA, as well as the physical function and knee pain of individuals [10]. Moreover, muscle mass in the lower limbs is independently influenced by the presence of KOA [11] and related to the severity of symptoms in patients [12].

The main approach to improve muscle strength and mass is resistance training (RT). RT can decrease the risk of KOA by increasing muscle strength and muscular hypertrophy [13]. Based on the recommendations of the American College of Sports Medicine, a resistance load of 60–80% of an individual’s one-repetition maximum (1RM) is necessary to achieve muscle hypertrophy and improvements in muscular strength [14]. However, high-load resistance training (HLRT) can aggravate pain and joint deterioration in patients [15], resulting in decreased compliance to therapeutic exercises and a slower rehabilitation process.

Blood flow restriction (BFR) training has drawn the attention of clinicians and physiotherapists in the field of musculoskeletal rehabilitation, as it is believed to be an alternative approach to HLRT. In brief, a pneumatized cuff or tourniquet is used to block partial arterial blood flow to the limb, and this is combined with 20–30% 1RM low-load resistance training (LLRT). By creating a state of ischemia in the limbs, BFR training will produce a stronger physiological metabolic stress that includes growth hormones [16, 17] and increased recruitment of type II muscle fibers [18]. Thus, even though the load is relatively low during BFR training, it can still bring increases in muscle strength and mass, similar to the results obtained by HLRT. More importantly, BFR training requires a lower external load, which is beneficial in decreasing the joint loading and tolerance of RT.

Previous studies have found that BFR combined with LLRT yields better improvements in muscle strength compared with LLRT alone in patients with a risk of KOA [19, 20], while its effectiveness in increasing muscle strength, muscle mass, and function in patients with KOA is similar to that of HLRT [21, 22]. Nonetheless, it remains unclear whether the degree of BFR will influence the effectiveness of BFR with LLRT. Many factors contribute to the degree of BFR, including systolic blood pressure, diastolic blood pressure, sex, limb circumference, limb length, and cuff width [23, 24]. The application of limb occlusion pressure (LOP) can produce a more precise degree of arterial occlusion for different individuals compared with absolute air pressure. Only one previous trial has used the percent of LOP to identify the pressure for BFR training in patients with KOA [22]. Notably, 70% LOP BFR training has a similar effect in increasing muscle strength, quadriceps muscle mass, and function compared with HLRT. However, it has been shown that BFR pressures with 40% LOP are as effective as 80% LOP [15] in the acute response of muscles. Nonetheless, the ability to achieve a chronic response with BFR remains unclear, especially for patients with KOA. Additionally, while a higher LOP promotes more pain during exercise with BFR training [25], it seems that pressure with 40% LOP may be more comfortable for individuals compared with 80% LOP, making it beneficial in eliciting compliance to the intervention program.

It has shown in previous studies that the effectiveness of BFR with LLRT is not conclusive compared with LLRT alone [26]. For instance, Ferraz et al. demonstrated that LLRT with BFR is more effective in relieving pain compared with LLRT alone [22], while another study suggested that there was no difference between the two approaches [19]. Therefore, in this study, we set a group of LLRT without BFR to investigate whether LLRT with lower LOP is more effective than LLRT alone. Due to the fact that health education constitutes a minimized intervention that is nonetheless important for KOA [27], we set health education as a control group for comparison with the other three groups. The aim of this study is to investigate the efficacy of LLRT with different degrees of BFR in patients with KOA. Our measures will include pain, self-reported function, physical functional performance, muscular strength, muscle thickness, and QoL. We will also observe any adverse events to verify the safety of BFR training during the study.

Hypotheses:

1. LLRT, LLRT with low BFR (40% LOP), and LLRT with high BFR (80% LOP) will all be more effective than health education.
2. Both LLRT with 40% BFR and 80% BFR will be more effective than LLRT alone, but there will be no difference between the two levels of BFR.

## Methods

### Study design

**Fig. 1:**
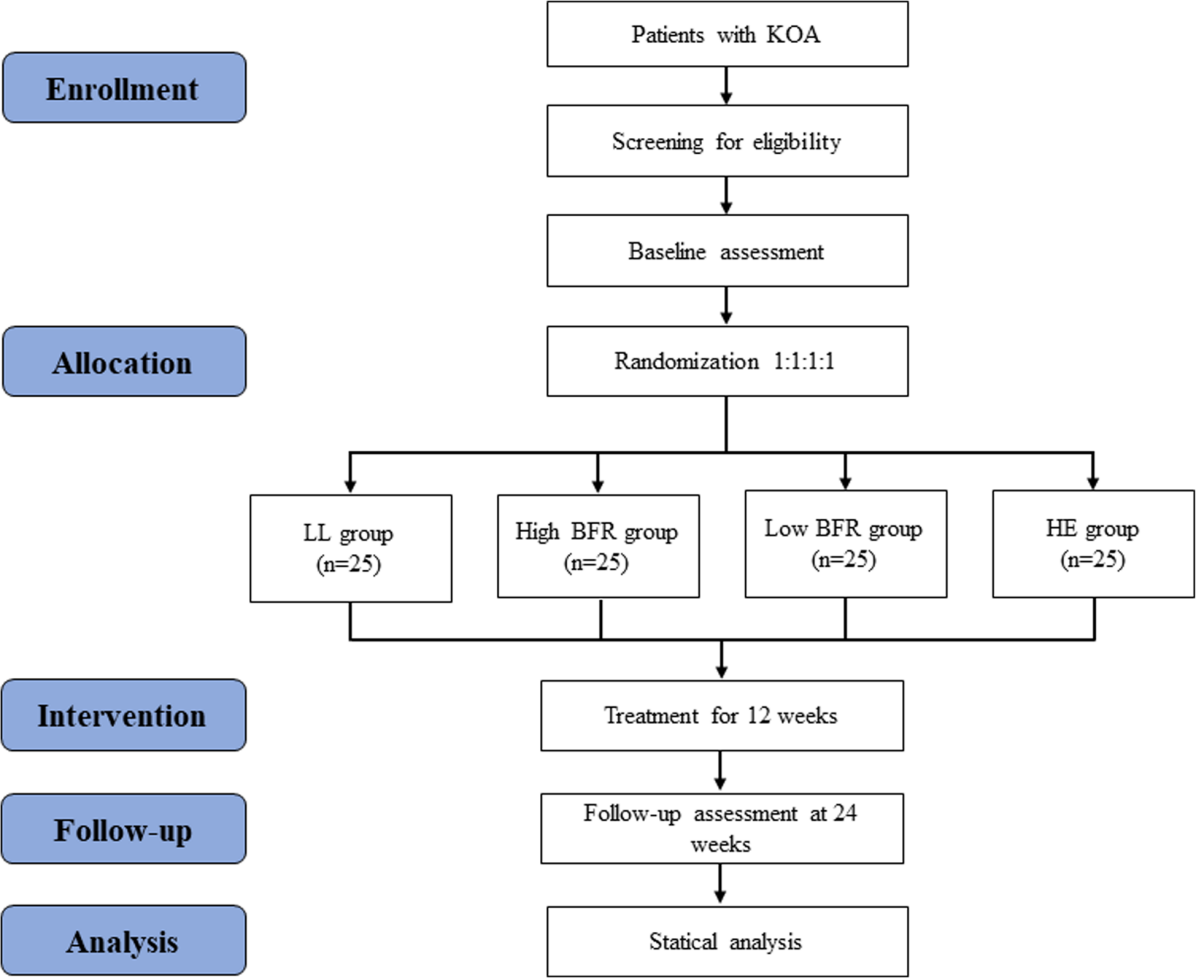
Flow diagram of the planned study. KOA, knee osteoarthritis; LL, low load; BFR, blood flow restriction; HE, health education

**Fig. 2:**
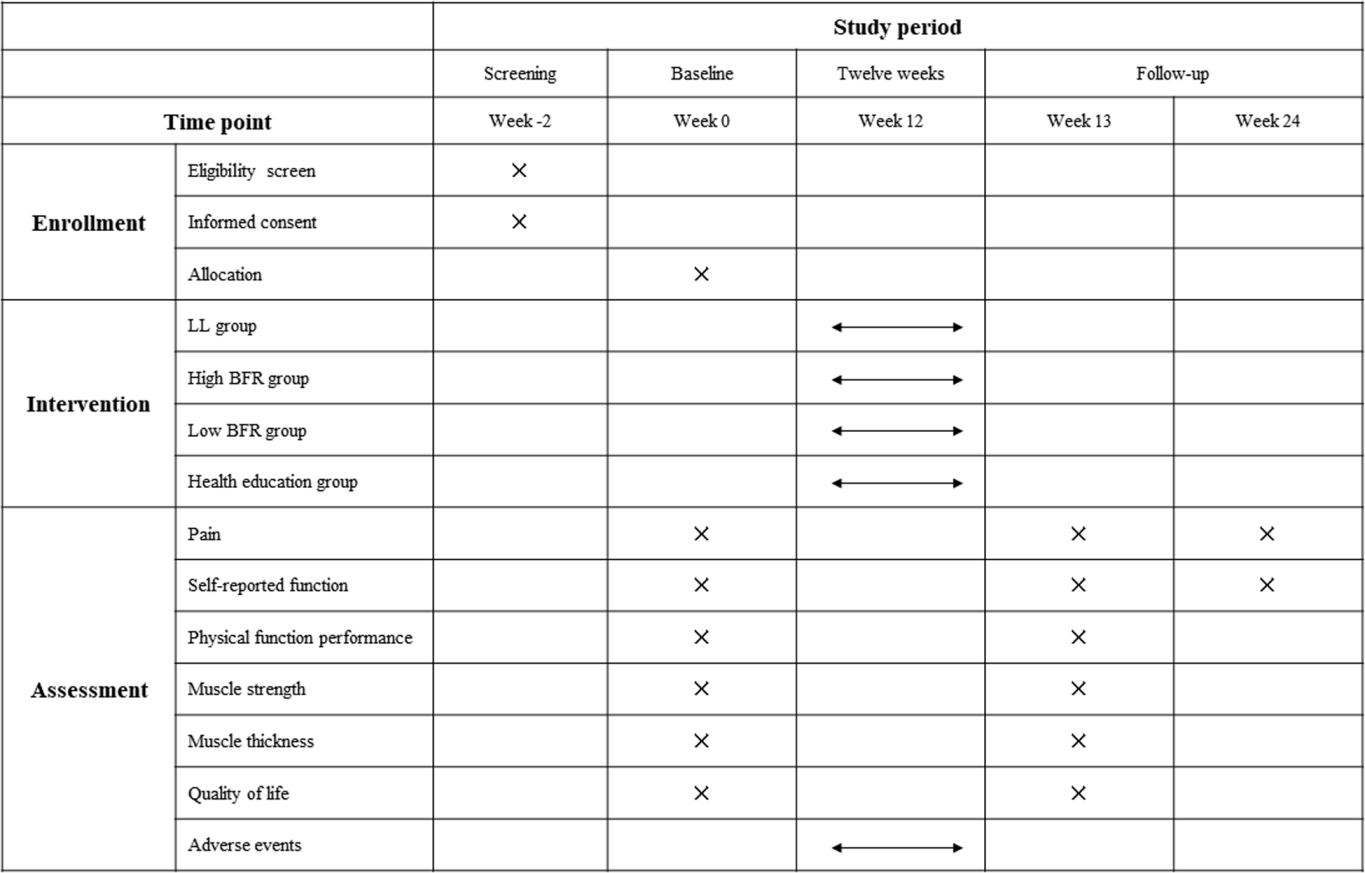
Schedule of enrollment, intervention, and assessment (SPIRIT figure). LL, low load; BFR, blood flow restriction

This study will be a prospective, single-blind, randomized controlled trial conducted in Beijing Chaoyang Hospital. The measurements of several outcomes will be made before and after the interventions and 24 weeks after the interventions begin (Fig. 1). This study was approved by the Ethics Committee of Beijing Sport University (Ethics Approval No. 2020108H) and registered at the Chinese Clinical Trial Registry (ChiCTR2000037859). We designed this study using the Standard Protocol Items: Recommendations for Interventional Trials (SPIRIT) statement [28] (Fig. 2). The results will be reported in accordance with the CONsolidated Standards of Reporting Trials (CONSORT) guidelines [29]. The participants will be informed that the trial will not collect any biological specimens for storage. This process will be carried out by researchers blinded to group allocation.

### Participants

In this study, we will recruit 100 male and female patients with a diagnosis of unilateral or bilateral KOA. For participants with bilateral KOA, the affected side with worse Kellgren-Lawrence grade will be identified as the affected leg. The participants will then be randomly assigned to different interventions. The recruitment of participants will conduct through targeted strategies, including social media (WeChat), websites, newspapers, and other community approaches. If an interested patient meets the eligibility criteria, then the informed consent process will be performed by researchers who are blinded to group allocation. All potential participants will be informed that the study will last for 6 months, that they have the right to withdraw at any time, and that the trial will not collect any biological specimens for storage. If they are uncertain whether they will be able to participate for the entire 6-month period, we will provide regular healthcare for their KOA rather than include them in the formal study. All participants will be asked to provide written informed consent before the intervention. Figure 1 demonstrates the flow chart for the trial, which includes participant recruitment, eligibility screening, baseline measurements, randomization and allocation, intervention, and outcome assessments. The enrollment of participants, the randomization procedure, and the performance of various measurements will be conducted by four independent physiotherapists. A manual will be developed to standardize the administration of the subjective questionnaires and the objective performance tests.

#### Inclusion criteria

The inclusion criteria will be as follows: (1) 45–75 years of age, (2) unilateral or bilateral KOA diagnosed according to the American College of Rheumatology clinical classification system [14, 30], (3) radiologic confirmation of KOA demonstrating Kellgren-Lawrence grade II or III [31], (4) average pain intensity of 40 or more on a 100-mm visual analogue scale (VAS) in the past week, and (5) adequate Mandarin language skills to complete the Western Ontario and McMaster Universities Osteoarthritis Index (WOMAC) and the written informed consent.

#### Exclusion criteria

The exclusion criteria will be as follows: (1) a history of knee surgery or scheduled surgery, (2) a history of any invasive procedure in the affected knee, including arthroscopy or intra-articular injection in the past 12 months, (3) a history of physical therapy/physiotherapy or a strengthening procedure of the affected knee in the past 6 months, (4) use of nonsteroidal anti-inflammatory drugs in the past 3 months, (5) any neurological, heart, or vascular disease, such as blood coagulation disorders, (6) abnormal blood pressure (resting systolic blood pressure [SBP] > 160 or < 100 mmHg or diastolic blood pressure [DBP] > 100 mmHg), or (7) other acute or chronic disorders or psychiatric conditions that will affect physical or cognitive functions.

### Withdrawal criteria and management

The KOA patients will be asked, or be allowed, to quit the study in the following cases: (1) the participants demand it and (2) there is a severe adverse event during the study.

### Randomization and allocation concealment

Eligible patients will be randomly assigned to one of the following groups at a ratio of 1:1:1:1: LL group, High BFR (80% LOP) group, Low BFR (40% LOP) group, and HE group. The research staff will independently use SAS software (SAS Institute, Cary, NC, USA) to generate a randomization sequence; this staff will not participate in the intervention or statistical analyses in the study. The number sequence will then be placed into a sequentially numbered, sealed, opaque envelope prior to the study by another independent research assistant who will also not participate in other parts of this study. Once the eligible patients with KOA complete the informed consent process, their demographic information and baseline measures will be recorded. Next, the research coordinator, who will not be involved in the measurement process, will have the authorization to open the envelopes in ascending order to determine a patient’s intervention group assignment.

### Masking

The participants will be informed that all of the interventions have proven efficiencies for their knee disorders, but it is still unknown which intervention works best. Moreover, they will be asked not to discuss their intervention content and group assignment at any time during the study. The intervention locations and times of intervention sessions will be separated for each individual. Unmasking will only be allowed in the case of severe adverse events and will be reported as part of the results of this study.

### Intervention

All participants in the intervention groups will complete 24 exercise sessions over 12 weeks, with two sessions per week, as described in Table 1. During the 12 weeks, the participants may continue their previous activities without aggravating their knee symptoms. In an individual exercise session, each group will perform a warm-up exercise to prevent injuries before the formal exercise protocol. Moreover, the pain intensity will be monitored using a VAS during the exercise, with some pain considered as acceptable. However, the load will be decreased by 20% if the pain intensity is higher than 20 mm/100 mm on the VAS [32]. During the period of intervention, all participants are not allowed to accept any other form of therapy, including medications.

**Table 1:** Treatment protocol performed by the LL group and the BFR groups

LL group and BFR groups	
• Hamstrings stretching, 3 repetitions of 30 s	
• Bridge with isometric contraction of the transversus abdominis-CORE training, 3 repetitions of 30 s^+^	
• Hip abduction with weights (side lying), 3 sets of 10 repetitions^+^	
• Calm exercises (side lying) with an elastic band, 3 sets of 10 repetitions^+^	
• Calf raises with weights (standing), 3 sets of 10 repetitions^+^	
• Sensori-motor training (standing) at mini-trampoline, 3 repetitions of 30 s	
• Leg press (machine), 0–60°, 3 sets of 15 repetitions*	
• Seated knee extension (machine), 90°–0° of knee flexion, 3 sets of 15 repetitions*	

*Load is 30% of the 1-repetition maximum

^+^The load will be adjusted every 4 weeks to maintain an effort of perception between 6 and 7 on the Borg scale

Each group will conduct a warm-up by cycling for 10 minutes before the LLRT or BFR training. The leg press will be performed between 0 and 60° of knee flexion, and the leg extension will be performed between 90 and 45° of knee flexion [33]. In addition to thigh muscle strength training, distal joint, proximal joint, and core muscle strength training will also be performed. This is because current evidence has shown that biomechanical changes occur in patients with KOA [34], and a combined multi-joint strength exercise is potentially more effective than knee strength training alone in knee musculoskeletal disorders [35]. To avoid possible injuries during the training, we will provide a relatively integrated therapeutic exercise program. Thus, stretching exercises and core training will also be adopted in this study. During the treatment, we will adopt the 10-point Borg scale to monitor the perceived effort for several exercises [36], as shown in the detailed exercise program in Table 1.

The exercise load will be individually set for each participant and adjusted every 4 weeks by re-evaluating the participant’s 1RM. Since a direct 1RM test could potentially cause knee pain or injury in patients with KOA, the 1RM will be estimated by performing a 7–10 RM test, which is the maximum load that an individual can complete for 7–10 repetitions. Previous studies have shown that the 7–10 RM test can accurately estimate the 1RM for leg press [37] and knee extension exercises [38]. The formula is as follows [39]: estimated 1RM = weight/(1.0278 - 0.0278 × reps).

#### Blood flow restriction exercise groups

All participants from the High BFR and Low BFR groups will be individually measured for the LOP and re-evaluated every 2 weeks during the study. The LOP will be measured with the participants in relaxed and supine positions. The portable color Doppler ultrasound (LOGIQ e, General Electric Company, Boston, USA) will be positioned at the ankle to measure the pedal pulse. A pneumatic cuff (7-cm width and 56-cm length) will be placed on the proximal thigh of the participant and inflated until the pedal pulse vanishes on the Doppler ultrasound. We will then slowly deflate the cuff, and when the pedal pulse returns, we will record the LOP. The Low BFR group will perform at 40% LOP, while the High BFR group will perform at 80% LOP during the exercise. The loads for the leg press and knee extension exercises will be 30% of the 1RM (estimated by 7–10 RM). Furthermore, participants in the High BFR and Low BFR groups will perform one set of 30 repetitions (or until exhaustion) and three sets of 15 receptions with a 30-s interval between sets [20]. The LOP will be adjusted every time to maintain a similar degree of BFR for each individual.

#### Low-load resistance exercise group

The LL group will conduct a sham BFR, in which the pneumatic cuff will be placed on the proximal thigh of the participant with adequate space for two or more fingers between the thigh and cuff. However, no pressure will be applied to the pneumatic cuff. The exercise protocol of the LL group is the same as the BFR group. The LL group will perform three sets of 15 repetitions with 30% 1RM and 30-s intervals between sets [19, 21, 32].

#### Health education group

The individuals in the health education group will attend sessions related to protecting their knee joints during daily life. The sessions will be conducted once a week for 12 weeks. Moreover, we will introduce the basic concept of KOA and the methods used to manage the risks of KOA through various articles from the Internet and leaflets.

### Outcome measures

#### Primary outcome measure

1. Pain

A VAS will be used to evaluate pain intensity at rest and under maximum situations during the last week. The VAS comprises a line with a length of 100 mm, in which “0 mm” corresponds to no pain and “100 mm” to the worst pain imaginable [40]. The VAS depicts pain effectively and is easily operated and widely applied in patients with KOA. The VAS will be evaluated at baseline and 12 and 24 weeks after the randomization of the participants. Notably, the minimal clinically important improvement for pain is about 20 mm/100 mm [41].

#### Secondary outcome measures

1. Self-report function

The knee function self-report outcomes will be measured using the WOMAC. This is a 24-item self-report questionnaire that assesses joint pain, stiffness, and physical functions related to KOA [42]. The maximum WOMAC score is 120, where a higher score indicates worse symptoms and functions of the knee. The Chinese version of the WOMAC has been shown to be both valid and reliable, as well as sensitive to changes in patients with KOA [43]. The WOMAC will be measured at baseline and 12 and 24 weeks after the randomization of the participants.

2. Physical function performance

The physical function performance will be assessed by the timed up and go (TUG) test [44]. Previous studies have found that the TUG test has a good intra- and inter-rater reliability (0.97 and 0.96, respectively) for KOA patients with Kellgren-Lawrence grades 1–3 [45]. During the test, the subject is timed and required to independently rise from an armchair that is 45 cm in height, walk forward following a straight line for 3 m, turn, walk back, and sit again. The average of three measurements will be used for analysis. The TUG will be measured at baseline and 12 weeks after the randomization of the participants.

3. Muscle strength

The quadriceps muscle strength will be evaluated by strength test of knee extension with an isokinetic test system (IsoMed 2000, D&R Ferstl GmbH, Hemau, Germany). Before the strength test, the participants will be fastened onto a dynamometric chair in a 90° sitting position, with the torso and thighs fixed by rigid belts. Then, the axis of the dynamometer will be adjusted in alignment with the center of the knee. Furthermore, the range of motion will be set individually for the participants by asking them to extend and flex their knees to maximum ranges. They will need to perform five constant flexion and extension motions using concentric contractions without a gravity-compensation model at three angular velocities of 60°/s, 90°/s, and 120°/s [46]. During the test, the participants will be encouraged to perform at their maximum effort [47]. The data will be recorded and calculated as the peak torque in Newton-meters, peak torque/body weight, and power in watts. Previous studies have demonstrated that the test–retest reliability for an isometric knee muscle strength assessment is 0.83 in patients with KOA [48]. The quadriceps muscle strength will be measured at baseline and 12 weeks after the randomization of the participants.

4. Muscle thickness

The muscle thickness of the quadriceps will be measured using a portable color Doppler ultrasound [32, 49]. Intra-rater and inter-rater reliabilities have demonstrated good validation when evaluating the muscle by ultrasound. Furthermore, the correlations between the ultrasound and magnetic resonance imaging (MRI) scans for muscle thickness of the vastus medialis, vastus lateralis, and rectus femoris are 0.86, 0.94, and 0.86, respectively [50, 51]. During the test, the probe will be placed at the mid-belly of these three muscles without depressing the skin. Each muscle will be measured from the adipose tissue–muscle interface to the muscle–bone interface three times. The images will be saved and then averaged. The size of the quadriceps will be estimated as the sum of these three muscles. The muscle thickness will be measured at baseline and 12 weeks after the randomization of the participants.

5. Quality of life

The 36-item Short Form Health Survey (SF-36) is a brief self-report questionnaire with 36 questions relevant to QoL in eight health dimensions, including emotional aspects, physical aspects, social aspects, vitality, bodily pain, general health, physical functioning, and mental health in psychometric properties. It can be summarized in two health scores, namely, physical and mental components, where higher scores indicate a better health condition. Previous studies have indicated that the Chinese version of the SF-36 is a relative and valid questionnaire for the general population. The SF-36 will be measured at baseline and 12 weeks after the randomization of the participants.

6. Adverse events

All adverse events will be recorded throughout the entirety of the trial. Patients will be made aware of potential adverse events during the consent process and instructed to notify a researcher when adverse events occur. Accidental injuries will be collected through systematic participant spontaneous reporting. Potential adverse events of BFR or RT include muscle soreness, knee pain, subcutaneous hemorrhage, and numbness. Additionally, physiotherapists and related specialists will categorize adverse events as treatment-related or not and monitor the severity of the adverse events within 24 h. We will report all adverse events and describe whether they are related to the study.

### Sample size estimation

Previous studies have shown that the effect size for pain is 0.38–0.49 [52, 53]. The sample size was estimated by using G-Power software (version 3.1.9.6, Heinrich-Heine-Universität Düsseldorf, Düsseldorf, Germany) with the following parameters: analysis of variance (ANOVA) with repeated measures, a type I error of 5% (*α* = 0.05), a power of 95% (*β* = 0.05), group numbers = 4, number of measurements = 3, and effect size = 0.38. The total sample size of this study should be a minimum of 84 participants. With a possible dropout rate of 15%, it is estimated that a sample size of 25 patients per group will be needed to verify our study hypotheses for the primary outcomes.

### Statistical analyses

All data will be expressed as mean ± standard deviation (SD). A linear mixed model with repeated measures will be run to assess the data for fixed factors of the trial (LL group, High BFR group, Low BFR group, and HE group) and time (primary and secondary outcomes). Additionally, two-way ANOVAs will be used to analyze the differences between baseline, post-intervention, and follow-up measurements. Data from all subjects will be included in the statistical analyses, and the ITT approach will be applied to avoid disrupting the randomization of groups from dropping out through the trial. Any subject who started treatment will be included in the final outcome analysis regardless of whether he/she completed the study. Additionally, the missing data will be multiply imputed using chained equations with predictive mean matching, imputing data for each group separately. Estimates from 10 imputed data sets were combined using Rubin’s rules [54]. The level of significance will be set at *P* < 0.05 for all data. Statistical analyses will be performed using SPSS 22.0 (SPSS Inc., Chicago, USA).

### Data management, monitoring, and quality control

The data will be carefully recorded by both printed and electronic case report forms (eCRFs). Only outcome assessors have access to the eCRFs, and all input data will be double-checked by two independent assessors. All data will be unmodifiable once input and checked through the eCRF. An independent Data and Safety Monitoring Board will be established from independent experts in orthopedics, physiotherapy, methodology, and statistics to review and interpret the trial data. The board will review the progress of the trial after 6 weeks and decide if premature closure of the study is required based solely on adverse events. The Institute of Sports and Health of China will be responsible for verifying the accuracy of the data. Only the statisticians will have access to the database to conduct final statistical analyses. The data collected from this trial will not be used in secondary or ancillary studies. In this trial, both online and on-site monitoring will be adopted to review the trial processes. The ethics committee of Beijing Sport University will monitor protocol violations weekly. The participants, ethics committee, and Chinese Clinical Trial Registry will be informed of any protocol modifications by email.

## Discussion

BFR training has become a novel intervention for lower limb degeneration or post-surgical musculoskeletal disorders. However, there have been no consistent recommendations for BFR training in treating KOA in any guidelines until now. In this trial, we will investigate the efficacy of LLRT combined with different BFR pressures (0%, 40%, and 80% LOP) on pain, self-reported function, physical functional performance, muscle strength, muscle mass, and QoL.

Although several studies have indicated that BFR combined with LLRT is more effective than LLRT alone [19, 20], the most efficacious degree of BFR has not been determined. If a 40% LOP during BFR will produce similar clinical outcomes as an 80% LOP during BFR in patients with KOA, resistance using a lower LOP can be a good choice for those who are unbearable for high degree of LOP. Additionally, there has been no study comparing the effects of BFR training with a control group without an exercise intervention, and a complete placebo control is not applicable for patients with KOA. Thus, we will set up a control group (the health education group), which will make the study more rigorous and objective. We believe our study will provide evidence for the effectiveness of RT with BFR, which is important for clinicians in treating patients with KOA.

This trial meets the requirements of methodology for the application of randomization, allocation concealment, and ITT approach, as well as masking for patients. In this trial, there will be two sessions per week for 12 weeks, yielding a total of 24 exercise sessions for the exercise intervention groups. We will investigate the short-term effects (12 weeks) of these four groups for all clinical outcomes and safety, as well as the mid-term (24 weeks) effects of pain and self-reported function.

We will use the percent of LOP to apply as similarly as possible the degree of BFR for individuals in each group. Moreover, the LOP will be tested before every session to avoid possible changes in the hemodynamics of the participants. Considering the potential placebo effect for patients in the LL resistance group, the BFR cuff will also be applied to the participants during the training, but the cuff will not be inflated. It is worth mentioning that only one study has monitored pain intensity during training and compared the differences between BFR training and HLRT [21]. In this study, we will evaluate the pain intensity during training compared to the tolerance to different degrees of BFR in patients with KOA.

Despite its strengths, this study has some potential limitations. First, due to the nature of the intervention, the physiotherapist cannot be fully masked during the research. Second, like other studies using BFR training, the LOP will be assessed at rest, and it may be altered by changes in body position and muscle contraction. Third, the intervention program consists of core training and stretching, in addition to quadriceps training, to provide better outcomes for patients with KOA. However, these interventions, especially the stretching exercises, may influence certain outcomes, such as muscle strength. At the end of this research, our results may provide more reliable evidence on the effectiveness of BFR training and identify the most appropriate degree of BFR in treating patients with KOA.

## Trial status

The currently approved version of the protocol is version 1.0 dated March 2021. Recruitment is still in progress and will be completed by February 2022.

## Data Availability

The data and the relevant results in this study will be shared through academic conferences and scientific papers. The datasets can be obtained from the corresponding author upon reasonable request.

## Abbreviations

KOA: Knee osteoarthritis
BFR: Blood flow restriction
LLRT: Low-load resistance training
HLRT: High-load resistance training
1RM: One-repetition maximum
LOP: Limb occlusion pressure
SD: Standard deviation
SF-36: Short Form 36
VAS: Visual analog scale
TUG: Timed up and go
WOMAC: Western Ontario and McMaster Universities Osteoarthritis Index
